# ECG-ViEW II, a freely accessible electrocardiogram database

**DOI:** 10.1371/journal.pone.0176222

**Published:** 2017-04-24

**Authors:** Young-Gun Kim, Dahye Shin, Man Young Park, Sukhoon Lee, Min Seok Jeon, Dukyong Yoon, Rae Woong Park

**Affiliations:** 1 Department of Biomedical Informatics, Ajou University School of Medicine, Suwon, South Korea; 2 Mibyeong Research Center, Korea Institute of Oriental Medicine, Daejeon, Korea; 3 Department of Biomedical Sciences, Ajou University Graduate School of Medicine, Suwon, South Korea; Ludwig-Maximilians-Universitat Munchen, GERMANY

## Abstract

The Electrocardiogram Vigilance with Electronic data Warehouse II (ECG-ViEW II) is a large, single-center database comprising numeric parameter data of the surface electrocardiograms of all patients who underwent testing from 1 June 1994 to 31 July 2013. The electrocardiographic data include the test date, clinical department, RR interval, PR interval, QRS duration, QT interval, QTc interval, P axis, QRS axis, and T axis. These data are connected with patient age, sex, ethnicity, comorbidities, age-adjusted Charlson comorbidity index, prescribed drugs, and electrolyte levels. This longitudinal observational database contains 979,273 electrocardiograms from 461,178 patients over a 19-year study period. This database can provide an opportunity to study electrocardiographic changes caused by medications, disease, or other demographic variables. ECG-ViEW II is freely available at http://www.ecgview.org.

## Introduction

Electrocardiograms (ECGs) provide valuable clinical information about a patient’s cardiac status. Since the widespread implementation of electronic health records (EHRs), ECG records and patient data–including laboratory test results and diagnosis of disease and prescribed drug histories–have accumulated in daily clinical practice. These records are an excellent source of practice-based evidence for evaluating electrophysiological changes on ECGs under many clinical circumstances.

Proarrhythmic changes in the ECG caused by adverse drug reactions are one of the most prevalent causes of drug withdrawal from the market. Some drugs are associated with life-threatening ECG changes, most notably torsade de pointes [1]. Cisapride may cause an acquired long QT syndrome, and the Federal Drug Administration has prohibited its sale in the United States [2]. Macrolides, quinolones, and nonsedating antihistamines have shown similar effects [3,4]. For this reason, the Federal Drug Administration has recommended evaluation of the proarrhythmic potential of drugs via “Thorough QT/QTc (heart rate-corrected QT) studies”.

However, there are some technical barriers to the implementation of such studies. Basic ECG management programs supplied by each vendor do not provide a method for transferring complete ECG parameters into individual hospital information systems. Additionally, many ECG records are still stored as printed documents or image files, from which numeric values cannot be simply extracted in digital form.

Other existing ECG databases (the STAFF III, Cardiac Safety Research Consortium ECG, and PhysioBank databases) are limited in that their data are obtained only from patients with specific medical conditions, or from certain studies and trials. The STAFF III database includes ECGs that were acquired from patients with myocardial infarction [5]. The Cardiac Safety Research Consortium ECG and PhysioBank databases include ECG data from clinical trials and drug safety studies, including thorough QT/QTc studies [6–9]. Because of their origin, these databases cannot include electrophysiological changes outside of specific circumstances.

In contrast to the above-mentioned databases, the previous ECG-ViEW database has no restrictions, except for a few constraints pertaining to meet the US Health Insurance Portability and Accountability Act Privacy Rule [10]. ECG-ViEW contains all diagnoses, drug prescriptions, and selected laboratory test results that can affect an ECG. It also contains ECG data from healthy people, for possible use as a reference cohort of the general South Korean population. From July 2012 to July 2016, there were many data requests (approximately 90) from 13 countries in Asia, North America, and Europe (S1 Table). In addition, two papers were published using the previous ECG-ViEW database [11,12]. However, ECG-ViEW contains only QT/QTc data; it does not provide the PR interval, QRS duration, and cardiac axis data. These can be used to examine electrophysiological changes in more diverse clinical circumstances. To compensate for this limitation, we re-extracted all of the ECG parameters.

The aim of this study was to establish a real-world ECG database that can be used to evaluate the effects of drugs and diseases on ECG changes, by updating and upgrading our previous ECG-ViEW database. This new database will provide an opportunity to evaluate the effects of a drug or combination of drugs, on electrophysiological changes in patients with many diseases and drug treatments. The new version of our ECG-ViEW database is the ECG-ViEW II.

## Materials and methods

### Database development

#### Data resources and patient characteristics

This study was performed using the standard 12-lead surface ECG data of one South Korean tertiary teaching hospital with 1,103 beds. The study protocol was approved by the Ajou University Hospital Institutional Review Board. All ECGs performed from 1 June 1994 to 31 July 2013 were included in the database. All numerical parameters were calculated using Marquette™ 12SL algorithms (versions 7, 13, and 22) developed by GE Healthcare. There were no restrictions with respect to comorbidities or prescribed drugs. The database contained 979,273 ECGs from 461,178 patients (Table 1). An average of 2.1 ECGs per patient were recorded in the database (S2 Table). A total of 188,823 patients received sequential ECGs; of those, 119,768 underwent more than two ECGs in 2 years (S1 Fig). The median and mean durations between the ECG recordings were 340 and 633 days, respectively (S3 Table). Among those patients, 119,768 patients had more than 2 ECG records within 2 years (S1 Fig). The median and mean number of drug prescriptions given to patients who had had at least two ECGs in a 2-year period were 9 and 20.18, respectively (S4 Table and S2 Fig). The average age of the patients was 42.6 ± 19.2 years, and male patients comprised 50.1% of all patients. The proportion of patients with South Korean ethnicity was 98.8%.

**Table 1:** Demographic and clinical characteristics of the ECG-ViEW II database population.

Characteristics		Value
Patients (n)		461,178
Healthy individuals		94,326
Number of patients according to department visited		
Outpatient		276,036
Inpatient		93,036
Emergency room visit		109,090
Electrocardiogram, n		979,273
Age, years		42.6 ± 19.2
Age categories in years, n (%) ^a^	0–9	31,020 (6.7)
	10–19	21,091 (4.6)
	20–29	49,033 (10.6)
	30–39	95,075 (20.6)
	40–49	94,534 (20.5)
	50–59	69,894 (15.2)
	60–69	55,635 (12.1)
	> 70	40,084 (8.7)
Male sex, n (%)		231,058 (50.1)
Age-adjusted CCI, n (%)	0	37,894 (8.2)
	1–2	32,403 (7.0)
	3–4	7,819 (1.7)
	5–6	156,614 (34.0)
	7–8	119,624 (25.9)
	≥9	106,824 (23.2)
ECG parameters	RR interval, ms	851.7 ± 197.0
	PR interval, ms	157.1 ± 26.7
	QRS duration, ms	91.1 ± 15.2
	QT interval, ms	390.0 ± 43.5
	QTc interval, ms	425.4 ± 31.5
	P axis (degrees)	48.4 ± 24.8
	QRS axis (degrees)	45.2 ± 38.1
	T axis (degrees)	46.2 ± 38.4
Source, n (%)	Electronic health records	48,083 (4.9)
	ECG management system	865,590 (88.2)
	Printouts	67,384 (6.9)
Department, n (%)	Emergency	173,356 (17.7)
	Health examination	177,972 (18.1)
	Inpatient	193,851 (19.8)
	Outpatients	435,878 (44.4)

Data are presented as means ± standard deviation or frequencies.

CCI, Charlson comorbidity index; ECG, electrocardiography

a. Age at the time when the first ECG was performed, before changed to the birth year group.

#### ECG data extraction

**Fig 1:**
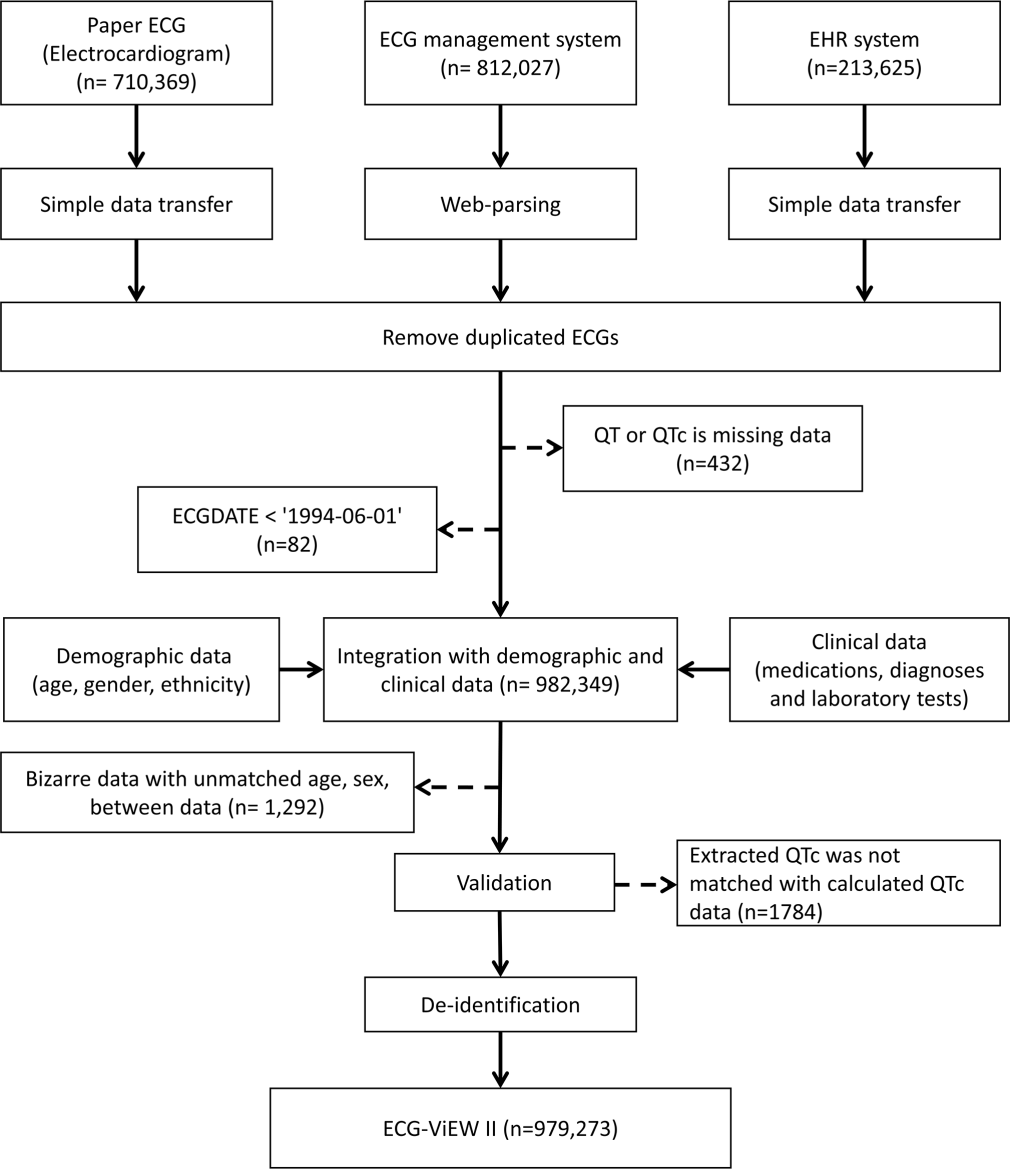
Schematic illustration of ECG-ViEW II construction. An overview of the database development process. All numeric ECG parameters were extracted from three ECG sources (ECG management system, EHR system, and ECG printouts) from the subject hospital. After remove duplicated ECGs, the ECG data were integrated with clinical data, validated with QTc data and de-identified. EHR, electronic health record; ECG, electrocardiogram; ECG-ViEW II, Electrocardiogram Vigilance with Electronic Data Warehouse II; QTc, heart-rate-corrected QT

The RR interval, QT/QTc interval, PR interval, QRS duration, P wave axis, QRS axis, and T wave axis values were extracted from each ECG data source. There were three sources from which the ECG data could be extracted: paper ECGs, digitalized ECG records in the ECG management system (MUSE; GE Marquette, Milwaukee, WI), and the EHR system. First, numeric ECG values on printed ECGs were copied from the previous ECG-ViEW database, which was originally extracted using optical character recognition. Second, we extracted PDF files from the ECG management system via the web-parsing software that was used in our previous work (but which was modified to extract additional parameters beyond those included in the previous version) [10]. All files in the system from 1 June 1994 to 31 July 2013 could be extracted automatically. Using the Java platform, the ECG parameters in the extracted PDF files were saved as text files. Finally, from 4 March 2010 to 31 July 2013, ECGs were recorded in a digitalized format in the EHR system of the target hospital. These records were simply transferred to our database (Fig 1).

The ECG data extracted by these three methods were merged into a single database after removal of duplicate data. The paper ECG data contained only the RR interval and QT/QTc interval. ECGs from both the ECG management system and the EHR contained the RR interval, QT/QTc interval, PR interval, QRS duration, P wave axis, QRS axis, and T wave axis. Thus, when the ECG management system data were duplicated with the EHR ECG data or paper ECG data, the ECG management system data remained and the other data were removed. Additionally, when the EHR data were duplicated with the paper ECG data, the EHR data remained and the paper ECG data were removed.

The ECG measuring devices in target hospital used Bazett’s formula (QTc = QT / [RR^0.5^]) to calculate the QTc interval. For this reason, the database contains QTc interval values calculated with Bazett’s formula. Other QTc data, based on Fridericia’s formula (QTc = QT / [RR^0.33^]) or Framingham’s formula [QTc = QT + 0.154 (1 − RR)], could be easily calculated using the QT and RR intervals provided in the database.

#### Extraction of demographic characteristics and clinical information

Using the EHR of the target hospital, we integrated the following demographic and clinical data into the ECG database: age, sex, ethnicity (Korean or non-Korean), prescribed medications, diagnoses, and selected serum electrolyte concentrations (potassium, magnesium, and calcium). The count of diagnosis included in the database is presented in the S5 Table. The observation period of a given patient was defined as the period from 1 year before the first ECG examination to 1 month after the last ECG examination. The age-adjusted Charlson comorbidity index was calculated with ICD-10 diagnosis codes, dating from 1994 to the date of the ECG.

#### De-identification

All unique identifiers were excluded to meet the US Health Insurance Portability and Accountability Act privacy rule. Outliers in the laboratory test results were replaced with a value at the 99.5^th^ percentile (top-coded). Highly stigmatized diagnoses (S6 Table), such as infertility, congenital malformation, sexually transmitted diseases (including HIV infection), and chromosomal abnormalities, were removed (215,725 diagnoses [2.7%] from 5,642 patients [1.2%]). Specific drugs that could be associated with, and used to identify, a specific person (especially antiviral agents for AIDS/HIV) were also removed. The examination date, drug prescription date, and diagnosis date were shifted by adding randomly assigned value within specific range (range: −90 to 90 days; mean, 0.0; standard deviation, 51.9). Thus, although an individual patient would not be identifiable using this information, the intervals between the dates for each individual patient were conserved. The birth years were grouped according to 5-year intervals (S7 Table).

### Software tools

We used Eclipse (ver. 3.2.2; IBM, Riverton, NJ) as a Java programming tool for the web-parsing and text-parsing software. MS-SQL 2014 (Microsoft, Redmond, WA) was used as the database management system.

### Code availability

The web-parsing software is available in our website: http://www.ecgview.org. The code is available under the terms of the GNU Affero general public license version 3 (https://www.gnu.org/licenses/agpl-3.0.html). This code was developed and tested using Eclipse (ver. 3.2.2; IBM, Riverton, NJ).

### Technical validation

Accuracy of the database was validated according to the correlation between QT and QTc. The QTc can be calculated according to QT intervals and RR intervals using Bazett’s formula. Extracted QTc values were compared with calculated QTc values. In total, 99.82% of extracted QTc values were matched to calculated QTc values. Data for which QTc values were not matched were excluded.

## Results and discussion

### Data records

The ECG-ViEW II database includes several ECG parameters (QT interval, QTc interval, and RR interval) that were already present in the previous version of the database; it also includes several additional parameters (PR interval, QRS duration, P wave axis, QRS axis, and T wave axis) that were not included in the previous database. ECG-ViEW II contains all of these parameters, which can be used to identify atrioventricular conduction abnormalities, diseases associated with a wide QRS complex, and diseases associated with cardiac axis deviation.

ECG-ViEW II contains about 20 years’ worth of data, which is over 2 years more than that of the previous ECG-ViEW database. It additionally contains about 270 thousand ECGs (total, 979,273), from 90 thousand patients (total, 461,178). The mean follow-up period per person was 554 ± 1,221 days; in comparison, the mean follow-up period in the first version of the database was 502 ± 1,008 days.

### Data tables

The database comprises seven tables (Person, Electrocardiogram, Drug, DrugcodeMaster, Diagnosis, DiagnosisCodeMaster, and Laboratory). A description of each table and its columns is provided in Table 2. Each table is associated with a randomly assigned patient identifier (“personid”).

**Table 2:** Overview of tables in the the ECG-ViEW II database.

Table name	Column name	Type, precision	Description
Person	personid	Integer	A randomly assigned patient identifier
	sex	Boolean	Patient sex; 1 = male, 0 = female
	birthyeargroup	Integer	Birthdates were grouped by 5-year intervals. The definition of each group is presented in S7 Table.
	ethnicity	Boolean	The state of belonging to an ethnic group; 1 = Korean, 0 = non-Korean
Electrocardiogram	personid	Integer	A randomly assigned patient identifier
	ecgdate	Date	The date on which the electrocardiogram was recorded
	ecgdept	Character (1)	Department in which the electrocardiogram was ordered E = Emergency, H = Health examination, O = Outpatient, I = Inpatient
	ecgsource	Character (1)	Origin of ECG data M = ECG management system, P = scanned paper ECG, E = electronic health records
	RR	Integer	RR interval, ms
	PR	Integer	PR interval, ms
	QRS	Integer	QRS duration, ms
	QT	Integer	QT interval, ms
	QTc	Integer	QTc interval, ms
	P_wave_axis	Integer	Degree of P wave axis
	QRS_axis	Integer	Degree of QRS axis
	T_wave_axis	Integer	Degree of T wave axis
	ACCI	Integer	Age-adjusted Charlson comorbidity index when ECG was performed
Drug	personid	Integer	A randomly assigned patient identifier
	drugdate	Date	Drug prescription date
	druglocalcode	Character (8)	Hospital electronic health record drug code
	atccode	Character (7)	Anatomical Therapeutic Chemical (ATC) drug code
	duration	Integer	Duration of drug use
	drugdept	Character (1)	Department that prescribed the drug (E = emergency, H = health examination, O = outpatient, I = inpatient)
	route	Character (1)	Route of drug administration (P = parenteral [injection], E = enteral)
DrugcodeMaster	druglocalcode	Character (8)	Hospital electronic health records drug code
	drugigrdname	Character (50)	Drug ingredient
Diagnosis	personid	Integer	A randomly assigned patient identifier
	diagdate	Date	The day on which the patient received a diagnosis code in the hospital
	diagcode	Character (100)	Diagnosis code with International Classification of Diseases (ICD) codes within observation period
	diaglocalcode	Character (8)	Diagnosis code with local electronic health record codes within observation period
	diagdept	Character (1)	Department in which diagnosis was made
DiagnosisCodeMaster	diaglocalcode	Character (8)	Diagnosis code with local electronic health record codes within observation period
	diagnosis	Character (190)	Diagnosis, full text
Laboratory	personid	Integer	A randomly assigned patient identifier
	labname	Character (1)	Name of laboratory test
	labdate	Date	Laboratory test performed date
	labvalue	Number (7,2)	Laboratory test value

### Usage notes

Before designing a study, researchers can download sample data, and an agreement form, from the ECG-ViEW II website (http://www.ecgview.org). This allows a quick understanding of our data structure. The agreement form contains basic provisions, stating for example that the researcher will not use the data for economic purposes, will not identify the patient, will not release the data to other people, etc. Proposals for the use of data in research and researcher’s valid certification number of the Collaborative Institutional Training Initiative (CITI) Program should be submitted to the Department of Biomedical Informatics of Ajou University School of Medicine, by using the application form on the website to obtain the complete raw data. After examining the submitted proposal, researchers can download the raw data from the ECG-ViEW II website in comma separated values file or mdf (for MS-SQL) or sql file (for MySQL). The ECG-ViEW II database is freely available to public researchers, but we prohibit our data from being used for commercial purposes.

### Collaborative research

#### Discovery of QT interval prolongation

Our previous ECG-ViEW database was developed to provide information on QT interval prolongation and associated factors [10]. The database is already being used to evaluate adverse drug reactions that result in QT interval prolongation. After examining the association of the QTc interval with certain drugs, Yun et al. [11] suggested that famotidine administration can prolong the QTc interval and increase the proarrhythmic potential. Additionally, Park et al. [13] concluded that selective serotonin reuptake inhibitors are less likely to be associated with QTc interval prolongation. The ECG-ViEW II contains previous ECG-ViEW data and an additional 270 thousand ECGs, allowing researchers to study the relationship between the QT interval and drug administration. According to Bazett’s formula, a QTc of > 450 ms in men, and > 470 ms in women, in considered abnormal[1]. Using these criteria, QTc prolongation was seen in 69,647 ECGs in men (n = 33,551) and 39,478 ECGs in women (n = 21,009) in the database (Table 3).

**Table 3:** Number of electrocardiograms and patients with abnormal ECG parameters.

	Electrocardiogram	Patients
Shortened PR interval	37,141	28,798
Prolonged PR interval	36,238	21,381
Wide QRS complex	27,680	12,096
Prolonged QTc interval	109,125	54,560
Abnormal P axis	88,819	61,467
Left axis deviation	27,173	12,222
Right axis deviation	39,637	25,528
Abnormal T axis	96,951	41,258

#### Discovery of conduction abnormality

The PR interval represents the time from the onset of atrial depolarization to the onset of ventricular depolarization. A shortened PR interval (< 120 ms) may be associated with Wolff-Parkinson-White syndrome or junctional rhythms. A prolonged PR interval (> 200 ms) may indicate a first-degree heart block, which is associated with a significant risk of atrial fibrillation (relative risk = 1.45), heart failure with left ventricular dysfunction (relative risk = 1.39), and mortality (relative risk = 1.24) [12]. The ECG-ViEW II contains data from 28,798 patients with a shortened PR interval and 21,381 patients with a prolonged PR interval (Table 3). The database also contains data on serum electrolytes, abnormalities of which can deteriorate PR interval prolongation. Analysis of PR interval data with electrolyte values allows researchers to study conduction abnormalities, heart failure, etc.

#### Discovery of diseases associated with cardiac axis deviation

ECG-ViEW II contains more information on the axis of P wave, QRS, and T wave than does our previous database. QRS axis deviation is the most important axis information. A normal QRS axis ranges from −30 to 90 degrees. A QRS axis of > 90 degrees is defined as right axis deviation. Right ventricular hypertrophy, chronic lung disease, lateral wall myocardial infarction, dextrocardia, and left posterior fascicular block can be associated with right axis deviation. A QRS axis less than −30 degrees is defined as left axis deviation, which can be associated with left ventricular hypertrophy, left bundle branch block, inferior wall myocardial infarction, Wolff-Parkinson-White syndrome, ventricular pacing/ectopy, and ostium primum atrial septal defect. The ECG-ViEW II database includes 39,637 ECGs showing right axis deviation (25,528 patients), and 27,173 showing left axis deviation (12,222 patients) (Table 3).

The normal P wave axis ranges from 0 to 75 degrees. Various studies have evaluated P wave axis deviations. Rangel et al. [14] showed that an abnormal P wave axis is correlated with atrial fibrillation. In addition, Acar et al. [15] showed that the P wave axis was significantly increased in patients with systemic lupus erythematosus and positively correlated with the SELENA-SLEDAI score. Another study showed that an abnormal P wave axis was associated with a 55% increased risk of all-cause mortality [16]. The ECG-ViEW II contains 88,819 ECGs with an abnormal P wave axis (61,467 patients) (Table 3). The database also contains all diagnoses of patients during the observation period. Researchers could evaluate the correlation of the P wave axis and diagnosis history.

The T wave axis was categorized as normal (15° to 75°), borderline (> 75° to 105° or −15° to < 15°), or abnormal (−180° to < −15° or > 105° to 180°). There was a nearly two-fold increased risk of coronary heart disease death, and an approximately 50% increased risk of incident coronary artery disease and all-cause mortality, for those with marked T wave axis deviation [17]. Moreover, T wave axis deviation is correlated with metabolic syndrome, low-grade systemic inflammation, and left ventricular hypertrophy in patient with diabetes mellitus or pregnancy [18–21]. ECG-ViEW II contains 41,258 patients with an abnormal T wave axis and provides an opportunity to evaluate the correlation of many diseases with the T wave axis parameter.

#### Discovery of cardiac disease in patients with a wide QRS complex

The QRS complex represents depolarization of the ventricles. A normal QRS complex duration is ≤ 120 ms in adults and ≤ 80 ms in children. An abnormal QRS complex may result from one of the following: an interventricular conduction disturbance, aberrant ventricular conduction, ventricular pre-excitation, and ventricular dysrhythmia. The ECG-ViEW II contains 27,680 ECGs with a wide QRS complex, from 12,096 patients (Table 3), thereby providing valuable information for researchers to investigate diseases associated with a wide QRS complex.

#### Discovery of correlation of ECG parameters with echocardiography parameters

An echocardiography database (EchoDB) was made after the previous ECG-ViEW database was opened to the public. It includes approximately 100,000 echocardiography data parameters from 72,399 patients. Among the parameters included in the EchoDB are the ejection fraction, left ventricular end diastolic and systolic dimensions, left atrium size, interventricular septal end diastolic and end systolic thicknesses, left ventricular mass index, ratio of mitral velocity to early diastolic velocity of the mitral annulus, and right ventricle systolic pressure. The ECG-ViEW II can be studied more thoroughly when combined with the EchoDB. If any researchers wish to evaluate the ECG data along with echocardiographic parameters, they can contact the ECG-ViEW II team in the Department of Biomedical Informatics in Ajou University (abmi@ajou.ac.kr). Researchers are required to send a study proposal (containing the study design) and complete the application form on the ECG-ViEW II website (send by email). After examining the submitted proposal and obtaining permission from the institutional review board of the target hospital, members of the ECG-ViEW II team analyze the data according to the researcher’s proposal.

### Strengths of the ECG-ViEW II database

One strength of the ECG-ViEW II database is that it contains real practice data. Compared with other ECG databases[5–9] that comprise data from clinical trials and specific medical circumstances, the ECG-ViEW II consists of real-world data of patients who have been prescribed drugs to treat many diseases. The ECG-ViEW II database allows researchers to evaluate the electrophysiological effects of drugs in many complicated and complex clinical situations.

Another strength of the ECG-ViEW II is that it consists of long-term follow-up data. The mean follow-up period was 554 ± 1,221 days; this increased from a mean of 502 days in the previous database. These long-term data allow researchers to evaluate both the short- and long-term electrophysiological effects of drugs. The ECG data can also be linked with the echocardiography data. Finally, the database is free to use. Any researchers who agree with our policy can use our database.

### Limitations of the ECG-ViEW II database

The database also has several limitations. First, the numerical parameters of the ECG were calculated using different algorithms. The ECGs in our database were collected over a period of 20 years, during which time the algorithms were upgraded. However, all ECG systems were obtained from GE Healthcare and approved by the United States Food and Drug Administration. Therefore, we are satisfied with the quality control of the algorithms. Second, it contains data from only one large hospital. Third, the age-adjusted Charlson comorbidity index of the database was calculated only within our observation period, and with the diagnostic data from only one hospital; thus, researchers should treat this score as reference rather than confirmative data. Last, no waveform data are provided. However, we have been collecting all biosignal data from the 30 intensive care unit (ICU) beds since August 2016, including ECG waveforms; arterial blood pressure; central venous pressure; and saturation, end-tidal CO2, and respiration curves. We plan to expand the ICU to 100 beds in 2017, and these data will be available to the public after we receive Institutional Review Board approval.

## Conclusion

The ECG-ViEW II database, described in this article, is a freely accessible electrocardiogram database. This database has integrated all numeric parameters of electrocardiogram, patient demographics, diagnosis data and drug prescription data. We believe that the ECG-ViEW II database will be an excellent data source for research scientists who study electrophysiological effect of diseases or drug prescription.

## Supporting information

S1 Dataset A subset of ECG-ViEW II dataset. (DOCX)

S1 Fig Histogram of the duration between ECG recordings in the same patient. (DOCX)

S2 Fig Histogram of the number of drug prescriptions per patient between ECG recordings. (DOCX)

S1 Table Number of downloads sorted by country and continent. (DOCX)

S2 Table Distribution of patients according to number of ECG recordings. (DOCX)

S3 Table Descriptive statistics of the duration between ECG recordings in the same patient. (DOCX)

S4 Table The number of drug prescriptions given to patients between ECG recordings. (DOCX)

S5 Table The number of times the diagnostic code was used in ECG-ViEW II database. (XLSX)

S6 Table Highly stigmatized diagnoses removed from the database. (DOCX)

S7 Table Birth year group code assignments. (DOCX)
