# Supplementary material for: ECG-ViEW II, a freely accessible electrocardiogram database
Source: PLoS One. 2017 Apr 24;12(4):e0176222. doi: 10.1371/journal.pone.0176222 (PMC5402933; doi:10.1371/journal.pone.0176222)
Supplement: S1 Dataset — (DOCX) [file pone.0176222.s001.docx]

**S1 Dataset. A subset of ECG-ViEW II dataset**

A subset ECG-ViEW II dataset is provided at <https://figshare.com/s/3ea30ba1ac39af387358>
