# Supplementary material for: ECG-ViEW II, a freely accessible electrocardiogram database
Source: PLoS One. 2017 Apr 24;12(4):e0176222. doi: 10.1371/journal.pone.0176222 (PMC5402933; doi:10.1371/journal.pone.0176222)
Supplement: S1 Fig — (DOCX) [file pone.0176222.s002.docx]

**S1 Fig. Histogram of the duration between ECG recordings in the same patient**


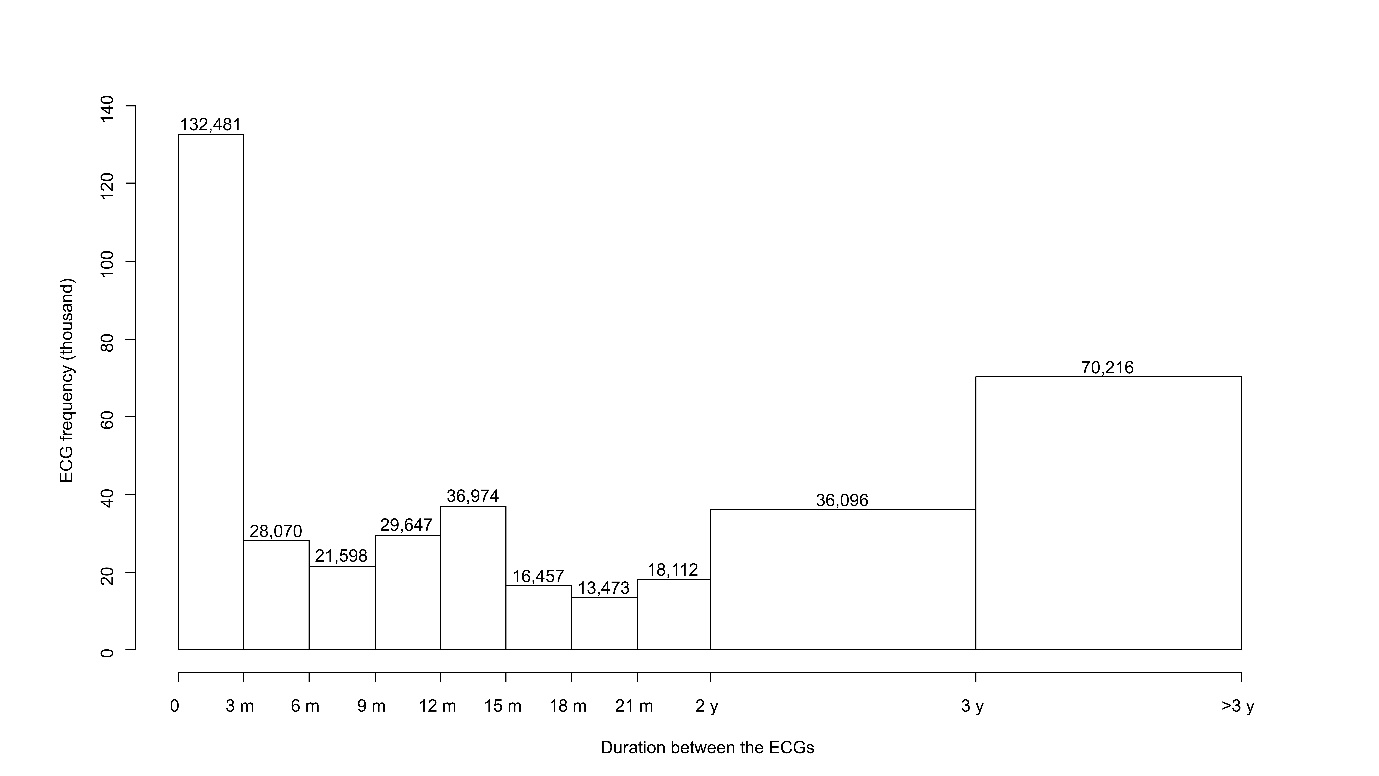


Abbreviations: m, months; y, years.
