# Supplementary figures and images for: ECG-ViEW II, a freely accessible electrocardiogram database
Source: PLoS One. 2017 Apr 24;12(4):e0176222. doi: 10.1371/journal.pone.0176222 (PMC5402933; doi:10.1371/journal.pone.0176222)

**S2 Fig. Histogram of the number of drug prescriptions per patient between ECG recordings**


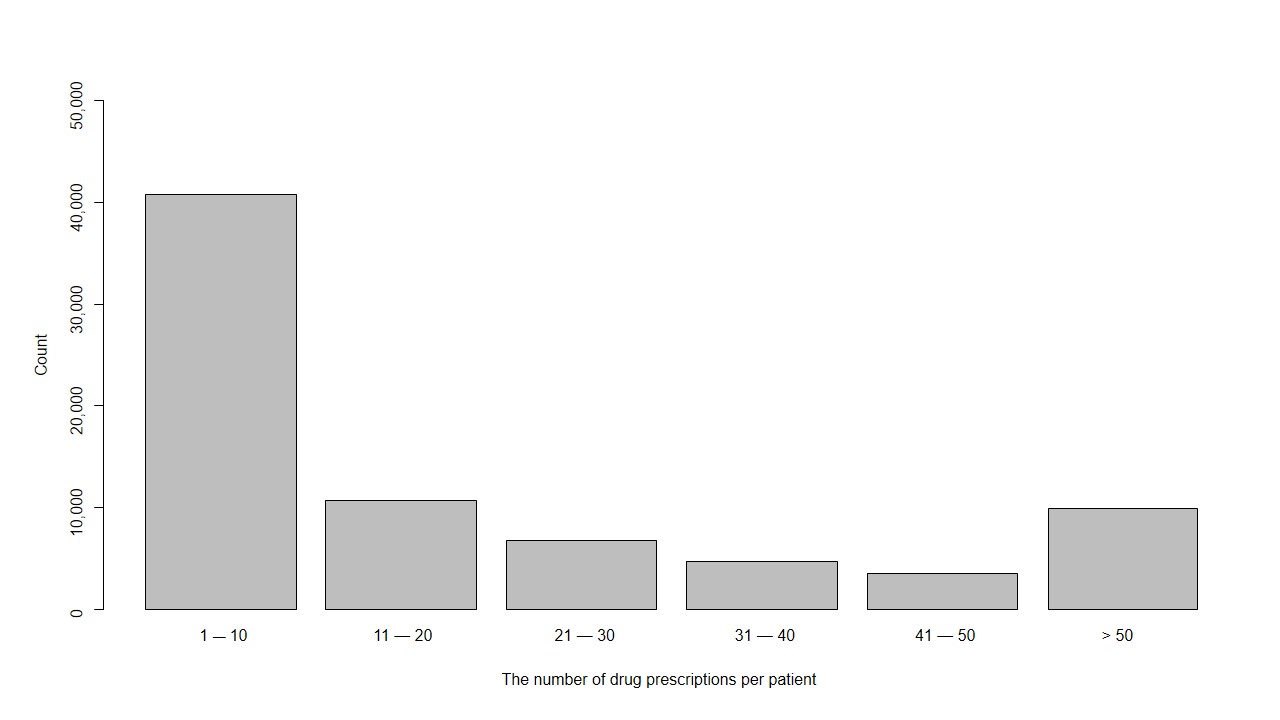

Supplement: S2 Fig — (DOCX) [file pone.0176222.s003.docx]
