# Supplementary material for: ECG-ViEW II, a freely accessible electrocardiogram database
Source: PLoS One. 2017 Apr 24;12(4):e0176222. doi: 10.1371/journal.pone.0176222 (PMC5402933; doi:10.1371/journal.pone.0176222)
Supplement: S1 Table — (DOCX) [file pone.0176222.s004.docx]

**S1 Table. Number of downloads sorted by country and continent**

| **Continent** | **Country** | **Number of downloads** |
| --- | --- | --- |
| **Asia** |  | **66** |
|  | Republic of Korea | 60 |
|  | India | 3 |
|  | Iran | 2 |
|  | Bangladesh | 1 |
| **North America** |  | **14** |
|  | USA | 13 |
|  | Canada | 1 |
| **Europe** |  | **11** |
|  | UK | 2 |
|  | France | 2 |
|  | Germany | 1 |
|  | Poland | 1 |
|  | Sweden | 1 |
|  | Slovenia | 1 |
|  | Russia | 1 |
|  | Ukraine | 1 |
