# Supplementary material for: ECG-ViEW II, a freely accessible electrocardiogram database
Source: PLoS One. 2017 Apr 24;12(4):e0176222. doi: 10.1371/journal.pone.0176222 (PMC5402933; doi:10.1371/journal.pone.0176222)
Supplement: S2 Table — (DOCX) [file pone.0176222.s005.docx]

**S2 Table. Distribution of patients according to number of ECG recordings**

| Number of ECGs | Patients (n) |
| --- | --- |
| 1 | 272,178 |
| 2 | 92,125 |
| 3 | 36,621 |
| 4 | 19,355 |
| 5 | 11,436 |
| 6–10 | 21,257 |
| 11–15 | 5,235 |
| 16–20 | 2,112 |
| 21–30 | 567 |
| 31–40 | 98 |
| 41–54 | 17 |
