# Supplementary material for: ECG-ViEW II, a freely accessible electrocardiogram database
Source: PLoS One. 2017 Apr 24;12(4):e0176222. doi: 10.1371/journal.pone.0176222 (PMC5402933; doi:10.1371/journal.pone.0176222)
Supplement: S3 Table — (DOCX) [file pone.0176222.s006.docx]

**S3 Table. Descriptive statistics of the duration between ECG recordings in the same patient**

|  | ECG duration |
| --- | --- |
| Minimum | 1 day |
| 25th percentile | 38 days |
| Median | 340 days |
| Mean | 633 days |
| 75th percentile | 756 days |
| Maximum | 6903 days |
