# Supplementary material for: ECG-ViEW II, a freely accessible electrocardiogram database
Source: PLoS One. 2017 Apr 24;12(4):e0176222. doi: 10.1371/journal.pone.0176222 (PMC5402933; doi:10.1371/journal.pone.0176222)
Supplement: S4 Table — (DOCX) [file pone.0176222.s007.docx]

**S4 Table. The number of drug prescriptions given to patients between ECG recordings**

|  | Drug prescriptions per person |
| --- | --- |
| Minimum | 1 |
| 25th percentile | 3 |
| Median | 9 |
| Mean | 20.18 |
| 75th percentile | 28 |
| Maximum | 247 |
