# Supplementary material for: ECG-ViEW II, a freely accessible electrocardiogram database
Source: PLoS One. 2017 Apr 24;12(4):e0176222. doi: 10.1371/journal.pone.0176222 (PMC5402933; doi:10.1371/journal.pone.0176222)
Supplement: S6 Table — (DOCX) [file pone.0176222.s009.docx]

**S6 Table. Highly stigmatized diagnoses removed from the database**

| ICD-10 codes | | Descriptions | |  |
| --- | --- | --- | --- | --- |
| A50-A64 | Infections with a predominantly sexual mode of transmission | |  |  |
| B20-B24 | Human immunodeficiency virus infection | | | |
| F10-F19 | Mental and behavioral disorders due to psychoactive substance use | | | |
| F52 | Sexual dysfunction not caused by organic disorder or disease | | | |
| F65 | Disorders of sexual preference | | | |
| F70-F79 | Mental retardation | | | |
| N46 | Male infertility | | | |
| N48 | Other disorders of penis | | | |
| N50 | Other disorders of male genital organs | | | |
| O00-O08 | Pregnancy with abortive outcome | | | |
| O30-O48 | Maternal care related to the fetus and amniotic cavity, and possible delivery problems | | | |
| Q00-Q07 | Congenital malformations of the nervous system | | | |
| Q10-Q18 | Congenital malformations of eye, ear, face, and neck | | | |
| Q20-Q28 | Congenital malformations of the circulatory system | | | |
| Q30-Q34 | Congenital malformations of the respiratory system | | | |
| Q35-Q37 | Cleft lip and cleft palate | | | |
| Q38-Q45 | Other congenital malformations of the digestive system | | | |
| Q50-Q56 | Congenital malformations of genital organs | | | |
| Q60-Q64 | Congenital malformations of the urinary system | | | |
| Q65-Q79 | Congenital malformations and deformations of the musculoskeletal system | | | |
| Q80-Q89 | Other congenital malformations | | | |
| Q90-Q99 | Chromosomal abnormalities, not elsewhere classified | | | |
| T73 | Effects of other deprivation | | | |
| T74 | Maltreatment syndromes | | | |
| X60-X84 | Intentional self-harm | | | |
| X85-Y09 | Assault | | | |
| Z72 | Problems related to lifestyle | | | |
| Z80-Z99 | Persons with potential health hazards related to family and personal history and certain conditions influencing health status | | | |
