# Supplementary material for: ECG-ViEW II, a freely accessible electrocardiogram database
Source: PLoS One. 2017 Apr 24;12(4):e0176222. doi: 10.1371/journal.pone.0176222 (PMC5402933; doi:10.1371/journal.pone.0176222)
Supplement: S7 Table — (DOCX) [file pone.0176222.s010.docx]

**S7 Table. Birth year group code assignments**

| Birth year group | Patient birth year |
| --- | --- |
| 0 | 1890–1894 |
| 1 | 1895–1899 |
| 2 | 1900–1904 |
| 3 | 1905–1909 |
| 4 | 1910–1914 |
| 5 | 1915–1919 |
| 6 | 1920–1924 |
| 7 | 1925–1929 |
| 8 | 1930–1934 |
| 9 | 1935–1939 |
| 10 | 1940–1944 |
| 11 | 1945–1949 |
| 12 | 1950–1954 |
| 13 | 1955–1959 |
| 14 | 1960–1964 |
| 15 | 1965–1969 |
| 16 | 1970–1974 |
| 17 | 1975–1979 |
| 18 | 1980–1984 |
| 19 | 1985–1989 |
| 20 | 1990–1994 |
| 21 | 1995–1999 |
| 22 | 2000–2004 |
| 23 | 2005–2009 |
| 24 | 2010–2013 |
